# Congenital Heart Disease and Genetic Changes in Folate/Methionine Cycles

**DOI:** 10.3390/genes15070872

**Published:** 2024-07-02

**Authors:** Nataša Karas Kuželički, Bojan Doljak

**Affiliations:** 1 Department of Clinical Biochemistry, Faculty of Pharmacy, University of Ljubljana, Aškerčeva 7, 1000 Ljubljana, Slovenia; 2 Department of Pharmaceutical Biology, Faculty of Pharmacy, University of Ljubljana, Aškerčeva 7, 1000 Ljubljana, Slovenia; bojan.doljak@ffa.uni-lj.si

**Keywords:** congenital heart disease, genetics, folate, methionine, transporters

## Abstract

Congenital heart disease is one of the most common congenital malformations and thus represents a considerable public health burden. Hence, the identification of individuals and families with an increased genetic predisposition to congenital heart disease (CHD) and its possible prevention is important. Even though CHD is associated with the lack of folate during early pregnancy, the genetic background of folate and methionine metabolism perturbations and their influence on CHD risk is not clear. While some genes, such as those coding for cytosolic enzymes of folate/methionine cycles, have been extensively studied, genetic studies of folate transporters (de)glutamation enzymes and mitochondrial enzymes of the folate cycle are lacking. Among genes coding for cytoplasmic enzymes of the folate cycle, *MTHFR*, *MTHFD1*, *MTR*, and *MTRR* have the strongest association with CHD, while among genes for enzymes of the methionine cycle *BHMT* and *BHMT2* are the most prominent. Among mitochondrial folate cycle enzymes, MTHFD2 plays the most important role in CHD formation, while FPGS was identified as important in the group of (de)glutamation enzymes. Among transporters, the strongest association with CHD was demonstrated for SLC19A1.

## 1. Introduction

Congenital heart defects (CHD) are one of the most common congenital malformations, occurring in approximately 9 per 1000 births [1]. There are many different subtypes of CHD, whose presentation varies from mild to life-threatening. There are several CHD classification systems, but the most informative for genetic studies is one that is based on embryological origins. In general, CHD can be divided into several broad classes: (1) conotruncal defects (double outlet right ventricle (DORV), transposition of the great arteries (TGA), persistent truncus arteriosus (PTA), tetralogy of Fallot (TOF), interrupted aortic arch (IAA)); (2) septal defects (ventricular septal defect (VSD), atrial septal defect (ASD)); (3) left ventricular outflow tract obstruction (LVOTO) (aortic stenosis (AS), coarctation of aorta (CoA), hypoplastic left heart syndrome (HLHS)); (4) right ventricular outflow tract obstruction (RVOTO) (pulmonary valve stenosis (PVS), pulmonary e atresia (PA), tricuspid atresia (TA), Ebstein anomaly); (5) atrioventricular septal defect (AVSD); and (6) anomalous pulmonary venous return (APVR) (total (TAPVR) or partial (PAVR)), heterotaxy and complex heart anomalies [2]. Patent ductus arteriosus (PDA) is technically speaking not a CHD, because it results from the failure of the fetal blood vessel (ductus arteriosus) to close shortly after the birth. However, because the genetic causes of PDA might be common to those of CHD, it is often studied together with CHD.

Although extensively researched, CHD risk factors are still unclear. In addition to genetic factors pertaining to the fetus, numerous environmental extrinsic and intrinsic factors related to the mother have been identified [3]. In the category of extrinsic factors fall factors that can be either an excess of a teratogen or the lack of an essential nutrient. Teratogens associated with congenital heart disease are thalidomide, retinoic acid, vitamin A, isotretinoin, alcohol, hypoxia (infants born at high altitudes), and various medicines (anticonvulsants, antiarrhythmics, antidepressants). Maternal nutritional deficiencies associated with CHD in offspring are folic acid deficiency and excess of homocysteine, vitamin B3 deficiency, and vitamin D deficiency. In some cases, the same substance might be considered an essential nutrient in low and teratogen in high quantities. For example, lack of or excess of retinoic acid might increase the risk of CHD development [4]. In the category of intrinsic factors fall maternal diseases and infections that change the uterine environment, such as maternal diabetes, obesity, phenylketonuria, viral infection (e.g., rubella), and hyperthermia [3].

Adequate intracellular folate levels are crucial for de-novo purine and thymidine synthesis and for the production of the essential amino acid methionine and elimination of the teratogenic homocysteine. It is also important for the synthesis of the main methyl donor S-adenosylmethionine, which is crucial to most remethylation reactions, including DNA methylation, an important regulator of gene expression during embryogenesis. The association of low folate levels and excess homocysteine with the development of neural tube defects (NTD) has been known for more than two decades. However, the association of lack of folate with congenital heart defects (CHD) has been established only recently [5]. Several large studies from China [6,7] and the Northern Netherlands [8] have found that maternal folic acid supplementation in the periconceptional period and during the first trimester of pregnancy have protective effects against CHD in the offspring. In contrast, the study on the large Danish National Birth Cohort and Norwegian Mother and Child Cohort Study found no such protective effect of folic acid supplementation in early pregnancy on CHD formation [9]. These opposing results might be due to the different pre-pregnancy folate statuses in different countries, which might be associated with dietary habits, and also to various confounders which are associated with folate-taking mothers and at the same time decrease the risk of CHD (lower body-mass index, less smoking, more pregnancy planning, lower parity, and a higher level of education). The reason for the opposing results might lie in the different observational time frames during pregnancy in different studies. Furthermore, the fetal environment regarding the available folate might be influenced by a maternal capacity to produce enough active folate forms, which is associated with the maternal genetic profile encompassing the genes of folate/methionine cycles. For example, a recent case-control study of mothers of CHD infants and mothers of healthy infants has found that two polymorphisms in the *MTHFR* gene (coding for an important enzyme of the folate cycle) in the mother increase the risk of CHD in offspring [10]. In recent decades, the genetic makeup of the fetus pertaining to the folate metabolism has been extensively researched. Variants in genes coding for folate influx and efflux transporters, enzymes associated with glutamation, folate, and methionine cycles in the cell cytoplasm, and mitochondria have been associated with CHD risk. While some genes have been extensively studied, others have been neglected. CHD-risk candidate genes and folate transport, as well as the identified associations with CHD development, are reviewed in the following sections.

## 2. Influence of Folates on Cardiac Neural Crest Cells

Neural crest cells are multipotent and highly migratory cell populations originating in the neural tube’s dorsal portion. During early fetal development, they undergo epithelial-to-mesenchymal transition, thus obtaining migratory potential, enabling them to disperse to different locations throughout the embryo, differentiating into various cell types. They are classified into four main cell subpopulations: cranial, vagal, trunk, and sacral neural crest. The vagal neural crest contains a smaller subpopulation of cells, called cardiac neural crest (CNC) cells, discovered in 1983 by Kirby and colleagues [11]. CNC cells significantly contribute to the development of the cardiovascular system but are also involved in the development of the thymus, thyroid gland, and cardiac ganglia [12,13,14,15]. CNC cells’ migration and differentiation are regulated by several signaling pathways, including BMP, Wnt, NOTCH, BAF, and Hippo-Yap [16].

CNC cells are highly responsive to folates through direct and indirect mechanisms. The direct impact of folates on CNC development is mainly through mitosis, DNA methylation, and folate receptors. Folate metabolism provides one-carbon groups for nucleotide synthesis. Adequate nucleotide synthesis is required for mitosis and is especially important in cells with high mitosis index, such as migratory CNC cells. Thus, CNC cells require high levels of methylated folate forms, and they consequently express high levels of folate receptors. Silencing the expression of folate receptors or lack of active forms of folate thus results in defective CNC cell migration and abnormal heart development [17,18,19]. Furthermore, low intracellular levels of active folate forms might lead to reduced DNA methylation [20], disrupting the expression of numerous genes [21,22], including those involved in heart development. Folates and folate receptors have a key role in the regulation of gene expression. The expression of many genes involved in CNC development changed in accordance with the folate levels during fetal development: *BMP4* [23], *GATA1* [24], *LRP6* [25], *NOTCH* genes [26], *CFL1* [27], *PAX3* [28], *PDGFRB* [29], *SHH* [30], *SMAD* [31,32], *VEGF* [33], *WNT* genes, and *CTNNB1* [34]. On the other hand, folate metabolism can exhibit an indirect effect on CNC development through the regulation of homocysteine levels. Lack of folate, vitamins B6 and B12, or genetic defects of genes of the folate/methionine cycle can result in the decreased re-methylation of homocysteine, thus increasing its intracellular levels. Homocysteine is a known teratogen working through four different mechanisms that can result in the disruption of CNC. Due to its thiol moiety, the homocysteine can increase the generation of reactive oxygen species, thus increasing oxidative stress [35]. Homocysteine might also alter the DNA methylation of the embryonic genome, which has a crucial impact on gene imprinting and embryonic development [36]. In high concentrations, homocysteine might cause N-homocysteinylation of lysine residues of various embryonic proteins, thus disrupting their function [37]. Lastly, homocysteine acts as an antagonist of NMDA receptors, which regulate neuronal development [38].

## 3. Folate Metabolism

**Figure 1:**
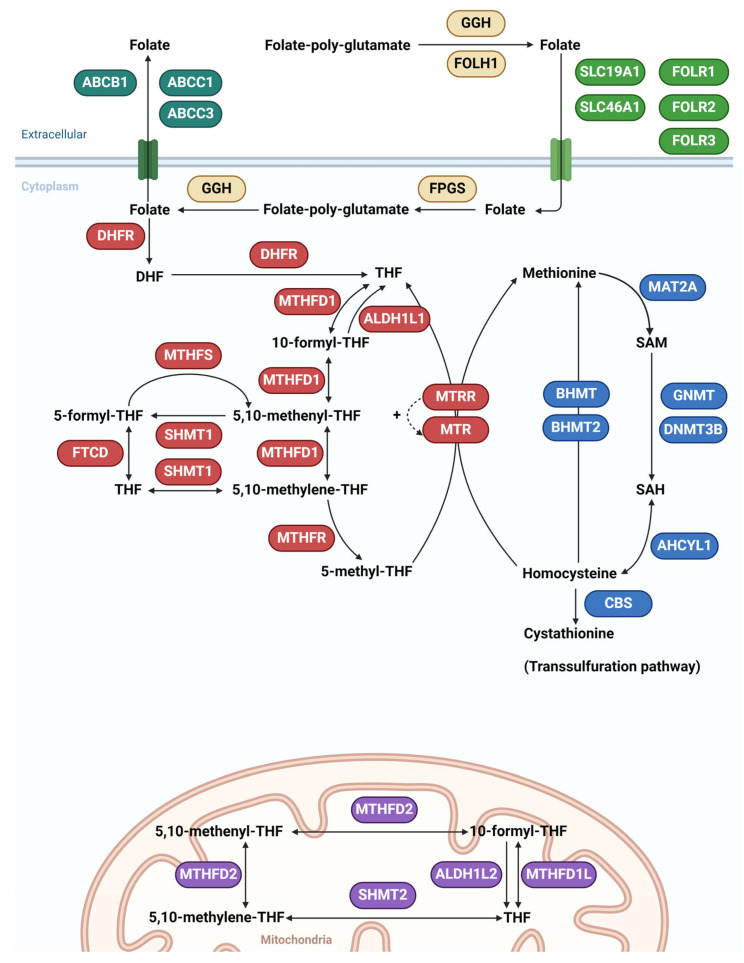
The folate and methionine metabolism in cytoplasm and mitochondria. Influx transporters are shown in green, efflux transporters in dark green, enzymes involved in glutamation in orange, cytoplasmic enzymes of the folate cycle in red, enzymes of the methionine cycle in blue, and mitochondrial enzymes of the folate cycle in purple. ATP Binding Cassette Subfamily C Member 1 (ABCC1), ATP Binding Cassette Subfamily C Member 3 (ABCC3), ATP Binding Cassette Subfamily B Member 1 (ABCB1), solute carrier family 19 member 1 (SLC19A1), solute carrier family 46 member 1 (SLC46A1), folate receptor α (FOLR1), folate receptor β (FOLR2), folate receptor γ (FOLR3), γ-glutamyl hydrolase (GGH), folate hydrolase 1 (FOLH1), folylpolyglutamate synthase (FPGS), dihydrofolate reductase (DHFR), methylenetetrahydrofolate dehydrogenase, cyclohydrolase and formyltetrahydrofolate synthetase 1 (MTHFD1), aldehyde dehydrogenase 1 family member L1 (ALDH1L1), serine hydroxymethyltransferase 1 (SHMT1), methenyltetrahydrofolate synthetase (MTHFS), formimidoyltransferase cyclodeaminase (FTCD), 5-methyltetrahydrofolate-homocysteine methyltransferase (MTR), 5-methyltetrahydrofolate-homocysteine methyltransferase reductase (MTRR), methylenetetrahydrofolate reductase (MTHFR), betaine-homocysteine S-methyltransferase (BHMT), betaine-homocysteine S-methyltransferase 2 (BHMT2), methionine adenosyltransferase 2A (MAT2A), glycine N-methyltransferase (GNMT), DNA methyltransferase 3 β (DNMT3B), adenosylhomocysteinase-like 1 (AHCYL1), cystathionine β-synthase (CBS), methylenetetrahydrofolate dehydrogenase 2, methenyltetrahydrofolate cyclohydrolase (MTHFD2), serine hydroxymethyltransferase 2 (SHMT2), methylenetetrahydrofolate dehydrogenase 1-like (MTHFD1L), aldehyde dehydrogenase 1 family member L2 (ALDH1L2), dihydrofolate (DHF), tetrahydrofolate (THF), S-adenosyl methionine (SAM), and S-adenosyl homocysteine (SAH), the dashed line represents the regeneration of MTR by MTRR.

Before entering cells, folate polyglutamates are hydrolyzed to folates (monoglutamates) by γ-glutamyl hydrolase (GGH). This is an important reaction since only the monoglutamate form can enter the cell through influx folate transporters. Folate hydrolase 1 (FOLH1) is important in the intestine, where it converts folate polyglutamate to folate monoglutamate, thus enabling its absorption from the gut to the blood. Folates are transported into cells by solute carrier family 19 member 1 (SLC19A1) transporter, a secondary active transporter that utilizes a gradient of organic phosphates to transport folates against their concentration gradient into the cell. Folate can also enter cells by solute carrier family 46 member 1 (SLC46A1) transporter, a symporter that utilizes a transmembrane H^+^ gradient. A third alternative is the transport of folates into the cell by the endocytic mechanism by folate receptor α (FOLR1), folate receptor β (FOLR2), and folate receptor γ (FOLR3). After entering the cell, folates are converted back to folate polyglutamates by folylpolyglutamate synthase (FPGS) thus preventing their exit from the cell. Only the monoglutamate folate form can exit the cell through the efflux transporters ATP Binding Cassette Subfamily C Member 1 (ABCC1), ATP Binding Cassette Subfamily C Member 3 (ABCC3) and ATP Binding Cassette Subfamily B Member 1 (ABCB1) (Figure 1).

Dihydrofolate reductase (DHFR) converts dihydrofolate (DHF) to tetrahydrofolate (THF). THF is then converted first to 10-formyl-THF, then to 5,10-methenyl-THF, and finally to 5,10-methylene-THF by enzyme MTHFD1, which possesses three distinct enzymatic activities: 5,10-methylenetetrahydrofolate dehydrogenase, 5,10-methenyltetrahydrofolate cyclohydrolase, and 10-formyltetrahydrofolate synthetase activity. Aldehyde dehydrogenase 1 family member L1 (ALDH1L1) has a MTHFD1 dehydrogenase activity and converts 10-formyl-THF to THF. 5,10-methenyl-THF and 5,10-methylene-THF can be converted by serine hydroxymethyltransferase 1 (SHMT1) to 5-formyl-THF and THF, respectively. Conversion of 5-formyl-THF to 5,10-methenyl-THF is catalyzed by methenyltetrahydrofolate synthetase (MTHFS), while interconversion between 5-formyl-THF and THF is catalyzed by formimidoyltransferase cyclodeaminase (FTCD). The central part of the folate cycle is the conversion of the 5,10-methylene-THF to 5-methyl-THF by methylenetetrahydrofolate reductase (MTHFR), the most extensively studied enzyme in this metabolic pathway. 5-methyl-THF is an active form of folate that serves as a methyl donor in the remethylation of homocysteine to methionine by 5-methyltetrahydrofolate-homocysteine methyltransferase (MTR), which in turn is regenerated to a functional state by 5-methyltetrahydrofolate-homocysteine methyltransferase reductase (MTRR). Remethylation of homocysteine to methionine is so important that it is catalyzed by several different enzymes using various methyl donors. The alternative remethylation reactions using betaine as a methyl donor instead of 5-methyl-THF are those catalyzed by betaine-homocysteine S-methyltransferase (BHMT) and betaine-homocysteine S-methyltransferase 2 (BHMT2) that are expressed ubiquitously and in the liver, respectively (Figure 1).

The methionine cycle is a key source of S-adenosyl methionine (SAM), which is the major methyl group donor in methylation reactions. Methionine adenosyltransferase 2A (MAT2A) catalyzes the transfer of the adenosyl group from ATP to methionine, resulting in SAM formation. After releasing its methyl moiety during various methylation reactions, SAM is converted to S-adenosyl homocysteine (SAH). This step is catalyzed by more than 60 different methyltransferases, but quantitatively most important are glycine N-methyltransferase (GNMT) and DNA methyltransferase 3 β (DNMT3B). Finally, SAH is converted to homocysteine by adenosylhomocysteinase-like 1 (AHCYL1). Homocysteine, which is toxic and teratogenic in high concentrations, is eliminated by the transsulfuration pathway, which begins with the conversion of homocysteine to cystathionine by cystathionine β-synthase (CBS) (Figure 1).

The folate metabolism is highly compartmentalized in eukaryotic cells. The folate cycle in mitochondria is catalyzed by the different sets of enzymes that are paralogs of the cytosolic enzymes of the folate cycle. MTHFD2 is a mitochondrial bifunctional enzyme with methylenetetrahydrofolate dehydrogenase and methenyltetrahydrofolate cyclohydrolase activities, while serine hydroxymethyltransferase 2 (SHMT2) is a mitochondrial form of a pyridoxal phosphate-dependent enzyme that catalyzes the reversible reaction of THF to 5,10-methylene-THF. It is paralog of SHMT1. Methylenetetrahydrofolate dehydrogenase 1-like (MTHFD1L) is a paralog of MTHFD1, and aldehyde dehydrogenase 1 family member L2 (ALDH1L2) is a paralog of ALDH1L1 (Figure 1) [39].

## 4. CHD Candidate Genes in Folate/Methionine Cycles

Through the informal literature review and databases search [39], we identified 31 candidate genes in folate/methionine cycles that might be theoretically associated with CHD based on their involvement in folate metabolism and transport. However, the association with CHD in humans was documented in published genetic studies on CHD patients for only 15 of 31 candidate genes. All the candidate genes and genes associated with CHD in humans are depicted in Table 1.

The highest number of candidate genes are located on chromosomes 5, 1, 17, and 21. Of note, a cluster of three genes involved in the transport of folate (SLC19A1), folate metabolism (FTCD), and homocysteine elimination (CBS) is located near the Down syndrome critical region on chromosome 21 (21q22.3). The incidence of CHD in Down syndrome cases has been reported to be as high as 40–60% and it represents the major cause of mortality in individuals with Down syndrome during the first years of life [40].

In the following sections, we review CHD candidate genes in folate/methionine metabolic pathways and their association with CHD.

## 5. Genes Coding for Folate Influx Transporters

Candidate genes *SLC19A1*, *SLC46A1*, *FOLR1*, *FOLR2*, and *FOLR3* were identified in this category, but only *SLC19A1* was shown to be associated with CHD in humans.

### 5.1. SLC19A1

SLC19A1 (previously known as reduced folate carrier 1, RFC-1) is an antiporter that mediates the import of reduced folates, driven by the export of organic anions. It has a high affinity for 5-methyl-THF, but can also transport antifolate drug methotrexate (MTX) and immunoreactive cyclic dinucleotides, such as cyclic GMP-AMP. Gene *SLC19A1* is located at 21q22.3 and has 29,876 bases, coding for 591 amino acids. Diseases associated with the mutations in this gene are folate-responsive megaloblastic anemia and immunodeficiency 114 [39].

The most frequently studied variant in the *SLC19A1* gene associated with congenital heart disease in humans is the missense mutation in the exon 2 of the gene, which results in an amino acid change on position 27 in the SLC19A1 protein; namely, histidine is replaced by proline (Table 2). The American College of Medical Genetics (ACMG) has set guidelines for the interpretation of DNA sequence variants, based on the population sequence of the variant and the pathogenicity predictions of several bioinformatic tools [41]. Using the ACMG criteria, this variant is classified as a variant of uncertain significance. Due to the fact that although it is extremely rare in healthy general populations, it is predicted as benign, neutral, or uncertain by most bioinformatic tools. In studies that differentiated patients by type of CHD, it is most commonly associated with conotruncal defects [42,43,44], while a recent meta-analysis on 724 children with CHD and 760 healthy children found that the presence of the wild type A allele increased the risk of developing CHD 1.36-fold (95% CI (1.06–1.75), *p* = 0.02) [45]. Finally, a study on Down syndrome patients with and without CHD revealed that several variants in SLC19A1 are associated with the increased risk of AVSD, with odds ratios between 1.34 and 3.78, depending on the SNP and genetic model. Interestingly, all the above-mentioned variants are in strong linkage disequilibrium (r^2^ ≥ 0.8) with rs1051266 (c.80A>G), indicating that rs1051266 is the most probable causal polymorphism. In contrast, two smaller case-control studies found no association between this variant and CHD [46,47]. This might be due to the small sample sizes of the two studies.

**Table 2:** A variant in the *SLC19A1* gene that is associated with congenital heart disease in humans.

Variant ID and Type	Population Allelic Frequency (GnomAD)	ACMG Classification	Associated CHD Phenotypes	References
rs1051266 NM_194255.4: c.80A>C ENST00000311124: p.His27Pro Missense	0.0004%	Variant of uncertain significance	Conotruncal	[42,43,44]
			CHD in general	[45,48]
			AVSD	[49]

Congenital heart disease (CHD), atrioventricular septal defect (AVSD).

### 5.2. SLC46A1

SLC46A1 is a proton-coupled folate symporter that mediates folate absorption using an H^+^ gradient as a driving force. It is also able to transport heme and antifolate drugs, such as methotrexate. The gene is located at 17q11.2 and has 12,556 bases, coding for 459 amino acids. Mutations in this gene have been associated with hereditary folate malabsorption and folate deficiency anemia [39]. So far, no variants in this gene have been associated with CHD.

### 5.3. FOLR1, FOLR2, and FOLR3

FOLR1, FOLR2, and FOLR3 are members of the folate receptor (FOLR) family and have a high affinity for folic acid and its reduced derivatives at neutral pH. They facilitate the delivery of 5-methyl-THF to the interior of cells by endocytosis. The amino acid sequence of these three transporters is very similar, with FOLR2 having a 68% and 79% sequence homology with the FOLR1 and FOLR3 proteins, respectively. The corresponding genes *FOLR1*, *FOLR2*, and *FOLR3* form a gene cluster on chromosome 11, namely at 11q13.4. *FOLR1* has 6615 bases, coding for 257 amino acids. Mutations in this gene have been associated with neurodegeneration due to cerebral folate transport deficiency. *FOLR2* has 5350 bases, coding for 255 amino acids. Mutations in this gene have been associated with neural tube defects and myelomeningocele. *FOLR3* has 4166 bases, coding for 245 amino acids. Mutations in this gene have been associated with the primary peritoneal carcinoma and choriocarcinoma of the testis [39].

It has been established that *FOLR1* knock-out mice suffer from congenital heart abnormalities, which were resolved by maternal folate supplementation in a dose-dependent manner. In addition, maternal *FOLR1* genotypes were found to be associated with folate rescue efficiency [19]. No variants in fetal *FOLR* genes have been associated with CHD risk in humans. However, maternal *FOLR1* and *FOLR2* genotypes of several genetic variants were recently found to affect CHD risk in offspring [50].

## 6. Genes Coding for Folate Efflux Transporters

Candidate genes *ABCB1*, *ABCC1*, and *ABCC3* were identified in this category, but only *ABCB1* was shown to be associated with CHD in humans.

### 6.1. ABCB1

ABCB1 is a member of the superfamily of ATP-binding cassette (ABC) transporters, the MDR/TAP subfamily. It has a wide range of substrates, including xenobiotics. The gene is located at 7q21.12, has 210,307 bases, and is coding for 1280 amino acids. Mutations in this gene are associated with colchicine resistance and inflammatory bowel disease 13 [39].

The silent mutation rs1045642 (Table 3) in exon 26 does not alter the amino acid sequence, nor the mRNA splicing, but influences the stability of mRNA, thus resulting in lower levels of the ABCB1 protein [51]. A more recent study, however, indicates that the new codon, which is less common in the human genome than the original one, can influence the translation rate and thus the ABCB1 protein folding, which in turn changes the substrate specificity of ABCB1 [52]. Since ABCB1 is an important efflux transporter for numerous xenobiotics (including medicines) and endogenous metabolites (including folates), there is an increased risk of CHD in children whose mothers are carriers of the rs1045642 synonymous variant and are taking medications in early pregnancy [53]. The risk was even higher in the absence of folate supplementation during pregnancy [54]. This variant was associated with an increased risk of septal defects, especially VSD [53].

**Table 3:** A variant in the *ABCB1* gene that is associated with congenital heart disease in humans.

Variant ID and Type	Population Allelic Frequency (GnomAD)	ACMG Classification	Associated CHD Phenotypes	References
rs1045642 NM_001348946.2: c.3435T>C NM_001348946.2: p.Ile1145= Silent	51%	Benign	CHD in general	[54]
			Septal defects (VSD)	[53]

Congenital heart disease (CHD), ventricular septal defect (VSD).

### 6.2. ABCC1

ABCC1 is a member of the superfamily of ATP-binding cassette (ABC) transporters, the MRP subfamily. It facilitates the export of organic anions and drugs from the cytoplasm and has a wide range of substrates, including xenobiotics. The gene is located at 16p13.11, has 194,120 bases, and is coding for 1531 amino acids. Mutations in this gene have been associated with several forms of deafness [39]. So far, no variants in *ABCC1* have been associated with CHD.

### 6.3. ABCC3

ABCC3 is a member of the superfamily of ATP-binding cassette (ABC) transporters, the MRP subfamily. It is involved in the transport of various substrates, including many drugs, toxins, and endogenous compounds across cell membranes. The gene is located at 17q21.33 and has 57,477 bases, encoding 1527 amino acids. Conditions associated with *ABCC3* variants include Dubin–Johnson syndrome and cholestasis [39]. No variants in *ABCC1* have been associated with CHD.

## 7. Genes Coding for Enzymes Involved in Folate (De)Glutamation

Candidate genes *FPGS*, *GGH*, and *FOLH1* were identified in this category, but only *FPGS* was shown to be associated with CHD in humans.

### 7.1. FPGS

FPGS (EC 6.3.2.17) is the enzyme that catalyzes the conversion of folates to polyglutamate derivatives. FPGS regulates the cellular retention of folates via their polyglutamylation. The *FPGS* gene is located at 9q34.11 and has 19,910 bases, coding for 587 amino acids. Mutations in this gene are associated with cutis laxa and subacute leukemia [39].

Variants in this gene were not extensively studied in connection to CHD. We found an association of intron variant rs1544105 (Table 4) in the *FPGS* gene with the LVOTO [43]. The variant is classified as benign by the ACMG due to its high frequency in the general population, its location in the non-coding region of the gene, and the lack of in silico predictions. This mutation may be in linkage disequilibrium with the causal mutation that was not investigated.

### 7.2. GGH

GGH (EC 3.4.19.9) catalyzes the hydrolysis of folylpoly-γ-glutamates and antifolylpoly-γ-glutamates by the removal of γ-linked polyglutamates and glutamate. *GGH* is located at 8q12.3 and has 24,527 bases, encoding 318 amino acids. Diseases associated with *GGH* include tropical sprue and pulmonary neuroendocrine tumors [39]. No *GGH* variants were associated with CHD in humans.

### 7.3. FOLH1

FOLH1 (EC 3.4.17.21) has both folate hydrolase and N-acetylated-α-linked-acidic dipeptidase activity and has a preference for tri-α-glutamate peptides. In the intestine, it might be required for the uptake of folate. In the brain, it modulates excitatory neurotransmission through the hydrolysis of the neuropeptide, N-aceylaspartylglutamate, thereby releasing glutamate. The gene is located at 11p11.12, has 63,547 bases, and is encoding 750 amino acids. Variants in *FOLH1* have been associated with hyperhomocysteinemia and lymph node carcinoma [39]. Up to now, only one study has investigated the possible association of *FOLH1* variants with CHD in humans but found no such connection [47].

## 8. Genes Coding for Cytoplasmic and Mitochondrial Enzymes of the Folate Cycle

This category has the highest number of candidate genes, namely nine genes coding for cytoplasmic (*DHFR*, *MTHFR*, *MTHFD1*, *MTR*, *MTRR*, *ADLH1L1*, *SHMT1*, *FTCD*, *MTHFS*) and four coding for mitochondrial enzymes (*MTHFD2*, *MTHFD1L*, *SHMT2*, *ALDH1L2*). Of these, genes for six cytoplasmic (*DHFR*, *MTHFR*, *MTHFD1*, *MTR*, *MTRR*, *MTHFS*) and one for mitochondrial enzyme (*MTHFD2*) were associated with the risk of CHD in humans.

### 8.1. DHFR

DHFR (EC 1.5.1.3) is a key enzyme of folate metabolism, reducing DHF into THF, which in turn enters the folate cycle. The *DHFR* gene has been mapped to 5q14.1, but several pseudogenes and *DHFR*-like genes have been found at different genomic locations. *DHFR* has 28,758 bases and codes for 187 amino acids. Mutations in this gene are associated with megaloblastic anemia and familial adenomatous polyposis 4 [39].

The studies on zebrafish knock-down and knock-in models revealed that the *DHFR* gene plays an important regulatory role in the process of heart development, and its copy number variations (CNVs) may be a part of the ethiopathological mechanisms of CHD [55,56]. However, studies on humans are scarce. The study by Zhu et al. [57] identified the polymorphism rs11951910 (Table 5), which had protective effects against conotruncal heart defects in Hispanic infants. This is an intronic polymorphism that was classified as benign by the ACMG due to its relatively high population frequency and benign prediction by the SpliceAI bioinformatic tool version 1.3.1 (released: 7 March 2020). Deletion of 19 nucleotides in intron 1 of the *DHFR* gene (Table 5) was found to be protective against CHD development in the Chinese population. This deletion is located in the regulatory region and affects the binding of the Sp1 transcription factor, thus influencing gene expression [47].

### 8.2. MTHFD1, MTHFD2, and MTHFD1L

MTHFD1 (EC 1.5.1.5, EC 3.5.4.9, EC 6.3.4.3) is a trifunctional enzyme that possesses three distinct enzymatic activities, 5,10-methylenetetrahydrofolate dehydrogenase, 5,10-methenyltetrahydrofolate cyclohydrolase, and 10-formyltetrahydrofolate synthetase. Each of these activities catalyzes one of three sequential reactions in the reversible interconversion of THF to 5,10-methylene-THF in the cytoplasm. The enzyme is coded by the *MTHFD1* gene located on chromosome 14 (14q23.3). *MTHFD1* has 75,427 bases, encoding 935 amino acids. Diseases associated with MTHFD1 include combined immunodeficiency and megaloblastic anemia with or without hyperhomocysteinemia and neural tube defects. An important paralog of this gene is *MTHFD1L* which codes for a mitochondrial enzyme with methenyltetrahydrofolate cyclohydrolase activity that converts THF to 10-formyl-THF in mitochondria. MTHFD1L (EC 6.3.4.3) provides the metabolic reaction required to link the mitochondria and the cytoplasm in the mammalian model of one-carbon folate metabolism thus complementing MTHFD2 (EC 1.5.1.15, EC 3.5.4.9), a mitochondrial bifunctional enzyme with methylenetetrahydrofolate dehydrogenase and methenyltetrahydrofolate cyclohydrolase activities. Both MTHFD1L and MTHFD2 are nuclear-encoded by the genes located on chromosomes 6 (6q25.1) and 2 (2p13.1), respectively. *MTHFD1L* has 236,209 bases and encodes 978 amino acids. Diseases associated with *MTHFD1L* include colon adenocarcinoma and Alzheimer’s disease 4. *MTHFD2* has 31,394 bases and is coding for 350 amino acids. Changes in this gene are associated with spina bifida and neural tube defects [39].

A recent study found an association of three polymorphisms (rs2236225, rs2236222, and rs11849530) (Table 6) in the *MTHFD1* gene with the risk of CHD, particularly VSD [58]. In the case of rs2236222, the variant allele was associated with an increased risk, and in the case of the other two polymorphisms with a decreased risk of CHD [58]. In concordance with these results, in one of our previous studies, we found that the variant allele of rs2236225 was associated with the decreased risk of conotruncal CHD and CHD in general. The risk was even greater in wild-type homozygotes of rs2236225 in the absence of maternal folate supplementation in pregnancy [43]. In contrast, Christensen et al. found that variant rs2236225 increases the risk of CHD, particularly tetralogy of Fallot and aortic stenosis [59]. The observed opposing effects of the rs2236225 variant allele might be because folate status may modulate the impact of the rs2236225 variant since the variant MTHFD1 enzyme has normal substrate affinity but decreased stability at 42 degrees C. Thermolability is reduced by magnesium adenosine triphosphate and eliminated by the folate compounds [59]. The levels of these modulators of MTHFD1 activity can greatly vary among populations and individuals [43]. Similarly to Christensen et al., Khatami et al. found an association between variant rs2236225 allele and increased risk of CHD [60]. Although most studies found an association between rs2236225 and CHD, some smaller case-control studies did not find a statistically significant difference between cases and controls in the frequency of the variant allele [42,47,61,62] Finally, in the Hispanic population in the USA, the *MTHFD1* variant rs11627387 (Table 6) was associated with an increased risk of conotruncal CHD [57]. Of the abovementioned variants, rs2236225 was most frequently studied in relation to CHD and other congenital malformations. This missense variant, located in exon 20 of the *MTHFD1* gene, is very common in the general population and is thus designated as likely benign by the ACMG. While most functional coding bioinformatic tools predict this variant to be benign (Revel, α Missense, Sift, Polyphen 2, MetaLR, PrimateAI, and BayesDel), MutationTaster and DANN designate it as deleterious. Variants rs2236222 and rs11627387 are located in the non-coding parts of exons 23 and 27 in *MTHFD1*, which are also transcription enhancer regions. The variants are classified as benign since they are quite common in the general population and were also predicted as benign by SpliceAI bioinformatic tool. Intronic variant rs11849530 was likewise classified as benign and is common in the general population.

The common variant rs828858 (Table 6) is located upstream of the *MTHFD2* gene and was designated benign by the ACMG. However, it was associated with an increased risk of CHD in a case-control study, including 620 CHD cases and 620 healthy children. In addition, this variant was specifically associated with some CHD subtypes, namely ASD, VSD, and PDA in additive (ASD and VSD) and recessive (PDA) genetic models [58]. These risks were further increased by the absence of maternal folate supplementation in early pregnancy [58]. No variants in the *MTHFD1L* gene have so far been associated with CHD in humans.

**Table 6:** Variants in the *MTHFD1* and *MTHFD2* genes associated with congenital heart disease in humans.

Variant ID and Type	Population Allelic Frequency (GnomAD)	ACMG Classification	Associated CHD Phenotypes	References
*MTHFD1*				
rs2236225 NM_005956.4: c.1958G>A NM_005956.4: p.Arg653Gln Missense	44%	Likely benign	VSD	[58]
			Conotruncal	[43]
			CHD in general	[43,59,60]
			TOF	[59]
			Aortic stenosis	[59]
rs2236222 NM_005956.4: c.2279+147A>G Non-coding transcript exon variant	9%	Benign	CHD in general	[58]
			VSD	[58]
rs11849530 NM_005956.4: c.2458-2060A>G Intron	21%	Benign	CHD in general	[58]
			VSD	[58]
rs11627387 NM_005956.4: c.2719-955G>A Non-coding transcript exon variant	28%	Benign	Conotruncal	[57]
*MTHFD2*				
rs828858 NM_006636.4: c.-3575T>A Intron, Upstream	35%	Benign	CHD in general	[63]
			ASD	[63]
			VSD	[63]
			PDA	[63]

Congenital heart disease (CHD), ventricular septal defect (VSD), tetralogy of Fallot (TOF), atrial septal defects (ASD), and patent ductus arteriosus (PDA).

### 8.3. MTHFR

MTHFR (EC 1.5.1.53) catalyzes the conversion of 5,10-methylene-THF to 5-methyl-THF, thus providing a cosubstrate for homocysteine remethylation to methionine. This is a key regulatory connection between the folate and methionine cycles. The *MTHFR* gene is located at 1p36.22 and has 20,733 bases, coding for 656 amino acids. Diseases associated with MTHFR include homocystinuria due to deficiency of MTHFR activity and schizophrenia.

The two most extensively studied polymorphisms in the context of folate deficiency-related CHD are rs1801133 and rs1801131 (Table 7), historically known as 677C>T and 1298 A>C, respectively. The rs1801133 missense polymorphism is located in the catalytical domain of the MTHFR enzyme and thus lowers its activity by 70% in homozygous mutants and by 30% in heterozygotes. Although its influence on enzyme activity has been proven in vitro and in vivo, it was classified as benign by the ACMG because it is very common in the general population. However, most of the bioinformatic tools predict this variant as deleterious. The rs1801131 missense variant is located in the regulatory domain of the enzyme, thus lowering its activity by 40% in mutant homozygotes and 20% in heterozygous individuals. The variant is very common in the general population and was predicted as benign by most bioinformatic tools, except for MutationTaster 2021 (released: 24 April 2021) and DANN which designated it as deleterious. The variant is classified as a variant of uncertain significance by the ACMG. rs1801133 and rs1801131 are in linkage disequilibrium, thus individuals with three or four variant alleles on the two loci are extremely rare in the general population. The compound heterozygotes on both loci have an enzyme activity that is comparable to rs1801133 variant homozygotes. Even though these two variants have been extensively studied for several decades in the context of various congenital malformations, the results of different studies are not in total agreement. This is not surprising since the design and quality of studies vary to a great degree. However, a recent umbrella review of systematic reviews and meta-analyses [64] provides solid proof that both variants increase the risk of CHD development. The authors included all meta-analyses on MTHFR variants and CHD that fulfilled the inclusion criteria up to July 2019, i.e., 11 for rs1801133 and 5 for rs1801131. Of 11 meta-analyses that investigated the rs1801133 variant, 7 found an association with CHD, while all 5 meta-analyses investigating rs1801131 found an association with CHD. Two meta-analyses on the subject that were published after July 2019 both found an association of rs1801133 with CHD [65,66]. However, Liu et al. [66] found that in the subgroup analysis, this association was present only in Asians, but not in Caucasian populations. An interesting recent study by Calzada-Dávila et al. found that epigenetics might play an important role in the penetrance of variant *MTHFR* alleles rs1801133 and rs1801131. The *MTHFR* gene was found to be hypermethylated in healthy individuals compared to the CHD patients, but only in healthy individuals who were carriers of variant alleles [67]. rs1801133 and rs1801131 were associated with various subtypes of CHD. While rs1801131 is most common in conotruncal defects, rs1801133 was associated with VSD, ASD, TOF, CoA, PVS, aortic valve stenosis (AVS), HLHS, and pulmonary atresia with intact ventricular septum (PA+IVS). PDA was associated with both variants (Table 7).

The silent variant rs2066470 (Table 7) was associated with an increased risk of CHD in the Chinese population [68]. This mutation has a benign ACMG classification due to the relatively high frequency in the general population and because it does not change the amino acid sequence of the MTHFR enzyme.

**Table 7:** Variants in the *MTHFR* gene associated with congenital heart disease in humans.

Variant ID and Type	Population Allelic Frequency (GnomAD)	ACMG Classification	Associated CHD Phenotypes	References
rs1801133 NM_005957.5: c. 665C>T * NM_005957.5: p. Ala222Val Missense	31%	Benign	CHD in general	[64,65,66]
			VSD	[69]
			PDA	[70]
			Conotruncal	[42]
			TOF	[61,71]
			PVS	[72,73]
			HLHS	[72]
			CoA	[72,74]
			AVS	[72]
			PA+IVS	[73]
			ASD	[70]
rs1801131 NM_005957.5: c.1286A>C ** NM_005957.5: p.Glu429Ala Missense	29%	Variant of uncertain significance	CHD in general	[64]
			PDA	[75]
			Conotruncal	[76,77,78]
rs2066470 NM_005957.5: c.117C>T NM_005957.5: p.Pro39= Silent	10%	Benign	CHD in general	[68]

Congenital heart disease (CHD), ventricular septal defect (VSD), tetralogy of Fallot (TOF), atrial septal defects (ASD), patent ductus arteriosus (PDA), pulmonary valve stenosis (PVS), hypoplastic left heart syndrome (HLHS), coarctation of the aorta (CoA), aortic valve stenosis (AVS), pulmonary atresia with intact ventricular septum (PA+IVS). * Historically c.677C>T. ** Historically c. 1298A>C.

### 8.4. MTR and MTRR

MTR (EC 2.1.1.13) catalyzes the final step in methionine biosynthesis. In the presence of MTR, the transfer of a methyl group from methylcobalamin (MeCbl) to homocysteine occurs, yielding enzyme-bound cobalamin and methionine in the cytosol. MeCbl, an active form of cobalamin (vitamin B12), is used as a cofactor for methionine biosynthesis. The cobalamin form is regenerated to MeCbl by a transfer of a methyl group from 5-methyl-THF. The processing of cobalamin in the cytosol occurs in a multiprotein complex composed of at least three enzymes, including MTRR (EC 1.16.1.8). MTRR is a key enzyme in methionine and folate homeostasis responsible for regenerating MTR to a functional state. The *MTR* and *MTRR* genes are located at 1q43 and 5p15.31, respectively. *MTR* has 26,019 bases coding for 1265 amino acids, while *MTRR* has 55,167 bases coding for 698 amino acids. Diseases associated with mutations in *MTR* and *MTRR* include homocystinuria-megaloblastic anemia and neural tube defects [39].

The most extensively investigated mutation in the *MTR* gene related to CHD risk is the missense variant rs1805087 (Table 8), located in exon 26. It is defined as a variant of uncertain significance by the ACMG, due to the high frequency in the general population. On the other hand, most of the bioinformatic tools found this mutation benign, except MutationTaster and DANN which classified it as deleterious. Although it has been associated with an increased risk of CHD in some studies [58], the results from different studies are opposing. Two meta-analyses failed to find the association between rs1805087 and CHD [65,79].

The intron variant rs1770449 (Table 8) is classified as benign by the ACMG due to high population frequency and benign prediction by the SpliceAI tool version 1.3.1 (released: 7 March 2020). In one study, it was associated with an increased risk of CHD. The risk was also increased in septal and conotruncal subgroup analyses [80].

rs1050993 (Table 8) is located in the 3 prime UTR region of *MTR*, which is a part of a gene that is transcribed to the mRNA but is not translated into the protein. It has a benign ACMG classification, as it is very common in the general population and is predicted to be benign by bioinformatic tools. Similarly, as rs1770449, it was associated with an increased risk of CHD, including septal and conotruncal CHD [80]. In fact, haplotype CAA (rs1770449-rs1805087-rs1050993) was associated with an increased risk of CHD [80].

The intron variant rs2275565 (Table 8) (ACGM classification benign) is common in the general population and predicted as benign by the SpliceAI tool. Together with rs1805087, it was associated with the increased risk of CHD in the Chinese cohort [63].

The regulatory region variant rs28372871 (Table 8) is located in the *MTR* promoter and thus decreases the transcription of the gene, leading to decreased enzyme levels and activity [81]. Although it was found to increase CHD risk, it is classified as benign by the ACMG. This is because of the high population frequency and benign predictions by bioinformatic tools.

The 3 prime UTR region variant rs1131450 (Table 8) is located at the miRNAs binding site within the untranslated region of the *MTR* gene. In the part of the gene sequence containing rs1131450, at least 3 important miRNA molecules bind and thus regulate *MTR* expression [81]. This variant was also associated with an increased risk of CHD and has a benign ACMG classification.

**Table 8:** Variants in the *MTR* and *MTRR* genes associated with congenital heart disease in humans.

Variant ID and Type	Population Allelic Frequency (GnomAD)	ACMG Classification	Associated CHD Phenotypes	References
	*MTR*			
rs1805087 NM_000254.3: c.2756A>G NM_000254.3: p.Asp919Gly Missense	20%	Variant of uncertain significance	CHD in general	[58]
rs1770449 NM_000254.3: c.2594+15T>C Intron	32%	Benign	CHD in general	[80]
			Septal	[80]
			Conotruncal	[80]
rs1050993 NM_000254.3: c.*1361A>G 3 prime UTR variant	73%	Benign	CHD in general	[80]
			Septal	[80]
			Conotruncal	[80]
rs2275565 NM_000254.3: c.2775+157G>T Intron	28%	Benign	CHD in general	[63]
rs28372871 NM_000254.3: c.-472G>T Promoter, regulatory variant	47%	Benign	CHD in general	[81]
rs1131450 NM_000254.3: c.*905G>A 3 prime UTR variant	28%	Benign	CHD in general	[81]
	*MTRR*			
rs1801394 NM_002454.3: c.66A>G NM_002454.3: p.Ile22Met Missense	47%	Variant of uncertain significance	CHD in general	[79,82,83,84,85,86]
			Conotruncal	[43]
			TOF	[44,85]
			Acyanotic	[87]
			ASD	[86]
			VSD	[44,86,88,89]
			PDA	[86]
rs1532268 NM_002454.3: c.524C>T NM_002454.3: p.Ser175Leu Missense	31%	Benign	CHD in general	[83,86]
			Acyanotic	[87]
			ASD	[86]
			VSD	[86,88,89,90]
rs326119 NM_002454.3: c.-26+755C>A Intron	56%	Benign	CHD in general, conotruncal CHD, septation defects, LVOTO, RVOTO, ASD, VSD, TOF	[91]

Congenital heart disease (CHD), ventricular septal defect (VSD), tetralogy of Fallot (TOF), atrial septal defects (ASD), patent ductus arteriosus (PDA), left ventricular outflow tract obstruction (LVOTO), right ventricular outflow tract obstruction (RVOTO), the asterisk (*) at rs1050993 and rs1131450 indicates that the mutations are located in the 3 prime UTR region.

The most extensively studied *MTRR* variant in association with CHD is rs1801394 (Table 8), a missense variant located in exon 2 of the gene. It is classified as a variant of uncertain significance according to the ACMG, since it is very common and was predicted to be benign by some bioinformatic tools, while Polyphen2 and DANN gave deleterious predictions. Three meta-analyses confirmed the association of this polymorphism with CHD [79,82,83], while Xu et al. [83] found the association only in Asians, but not in Caucasians. Of specific CHD subtypes the variant was most frequently associated with VSD [44,86,88,89] and TOF [44,85], but also with ASD and PDA in the Han Chinese population [86]. It was associated with acyanotic CHD in Iranians [87] and with conotruncal CHD in the Slovenian population [43]. However, two small case-control studies [47,62] and two family triad studies [76,92] found no association between the variant and CHD. This might be due to the small sample size or different study design in the case of family based studies.

Another well-known polymorphism, located in exon 5 of *MTRR*, is the missense variant rs1532268 (Table 8). It is classified as benign, due to high populational frequency and benign predictions by all functional coding bioinformatic tools. One meta-analysis confirmed the association of this variant with CHD, both in Caucasians and Asians [83]. Similar to rs1801394, this variant was most commonly associated with VSD [86,88,89,90], but also with ASD and PDA in the Han Chinese population [86], as well as acyanotic CHD in the Iranian population [87].

Intron variant rs326119 (Table 8) is very common in the general population and is predicted benign by bioinformatic tools. Thus, it has received a benign ACMG classification. However, both measurements of the *MTRR* mRNA levels in human cardiac tissue and in vitro luciferase assays in transfected cells show that rs326119 decreases *MTRR* gene transcription by changing the transcription enhancer binding site [91]. What is more, three case-control studies involving 2340 CHD cases and 2270 healthy controls from different parts of China demonstrated that the rs326119 variant is associated with the increased risk of CHD in general, but also with the increased risk of specific subtypes, namely conotruncal CHD, septation defects, LVOTO, RVOTO, ASD, VSD, and TOF [91]. Later on, this variant was also associated with neural tube defects, specifically anencephaly, as well as congenital malformations of the adrenal gland, demonstrating the multiple effects of this variant on human embryo development [93].

### 8.5. MTHFS

MTHFS (EC 6.3.3.2) catalyzes the irreversible conversion of 5-formyl-THF to 5,10-methenyl-THF, thus regulating carbon flow through the folate-dependent one-carbon metabolic network that supplies carbon for the biosynthesis of purines, thymidine, and amino acids. The location of the *MTHFS* gene is at 15q25.1. It has 63,795 bases coding for 203 amino acids. Diseases associated with *MTHFS* mutations include neurodevelopmental disorder with microcephaly, epilepsy, hypomyelination, and cutis laxa [39].

MTHFS has not been extensively studied concerning CHD. Nembhard et al. [94] found an increased risk of CHD in infants with *MTHFS* variant rs12438477 (Table 9) alleles, but only in the case of the maternal periconceptional use of selective serotonin reuptake inhibitors (SSRIs). On the other hand, Webber et al. [95] found an association between the fetal variant rs12438477 allele and increased risk of conotruncal CHD in mothers who reported no periconceptional folic acid supplementation, while in mothers who reported periconceptional folic acid supplementation, the same allele in infants was associated with the decreased risk of CHD.

### 8.6. SHMT1 and SHMT2

SHMT1 (EC 2.1.2.1) and SHMT2 (EC 2.1.2.1) are cytosolic and mitochondrial forms of serine hydroxymethyltransferase, respectively. This is a pyridoxal phosphate-containing enzyme that catalyzes the reversible conversion of serine and THF to glycine and 5,10-methylene-THF. This reaction provides one-carbon units for the synthesis of methionine, thymidylate, and purines in the cytoplasm, as well as the primary source of intracellular glycine. *SHMT1* and *SHMT2* genes are located at 17p11.2 and 12q13.3, respectively. *SHMT1* has 35,678 bases and encodes 483 amino acids, while *SHMT2* has 5363 bases and encodes 504 amino acids. *SHMT1* is located within the Smith–Magenis syndrome region on chromosome 17. It is also associated with adult acute lymphocytic leukemia and neural tube defects. On the other hand, *SHMT2* is associated with neurodevelopmental disorders characterized by cardiomyopathy, spasticity, and brain abnormalities [39].

*SHMT1* and *SHMT2* were not extensively studied in connection to CHD. Nembhard et al. found an association of the maternal *SHMT1* rs9909104 variant with an increased risk of CHD in offspring [94]. One study investigated the association of two *SHMT1* variants (rs638416 and rs117940726) in infants with CHD, but the results were inconclusive [96].

### 8.7. ALDH1L1 and ALDH1L2

ALDH1L1 (EC 1.5.1.6) and ALDH1L2 (EC 1.5.1.6) catalyze the conversion of 10-formyl-THF to THF in cytosol and mitochondria, respectively. *ALDH1L1* and *ALDH1L2* genes are located at 3q21.3 and 12q23.3, respectively. *ALDH1L1* has 94,433 bases coding for 902 amino acids, and its paralog *ALDH1L2* has 87,860 bases coding for 923 amino acids. Diseases associated with *ALDH1L1* include focal dermal hypoplasia and megalencephalic leukoencephalopathy with subcortical cysts 1, while diseases associated with ALDH1L2 include Sjogren–Larsson syndrome and succinic semialdehyde dehydrogenase deficiency [39]. So far, no study has investigated the association of variants in these two genes with CHD.

### 8.8. FTCD

FTCD (EC 2.1.2.5, EC 4.3.1.4) is a bifunctional enzyme that displays both transferase and deaminase activity. It channels one-carbon units from formiminoglutamate, a metabolite of the histidine degradation pathway, to the folate pathway. The *FTCD* gene is located in one of the Down syndrome critical regions on chromosome 21, i.e., 21q22.3. The gene consists of 19,420 bases and codes for 541 amino acids. Diseases associated with *FTCD* include glutamate formiminotransferase deficiency and autoimmune hepatitis [39].

*FTCD* was not extensively studied in connection with CHD. One genome-wide association study (GWAS) identified a variant in *FTCD* as a potential genetic marker of CHD, but this hit was not confirmed in an independent cohort [97]. Furthermore, there is a case report on an infant with CHD (DORV, VSD, TGA) with a microduplication of 21q22.3, a region where *FTCD* is located in addition to eight other genes. Although 21q22.3 is one of the Down syndrome-associated regions, the affected infant had no symptoms of Down syndrome except for CHD. The same microduplication was found in the phenotypically normal father [98].

## 9. Genes Coding for Enzymes of the Methionine Cycle

Seven candidate genes were identified in this category (*MAT2A*, *BHMT*, *BHMT2*, *GNMT*, *DNMT3B*, *AHCYL1*, and *CBS*). Almost all have been associated with CHD in humans, except *MAT2A* and *AHCYL1*.

### 9.1. BHMT and BHMT2

*BHMT* and *BHMT2* genes are located next to each other at 5q14.1. Their protein products are 73% identical [99], and they have the same enzyme activity: methylation of homocysteine to methionine, using betaine as a methyl donor. While BHMT (EC 2.1.1.5) is ubiquitously expressed, BHMT2 (EC 2.1.1.10) is expressed mostly in the liver. *BHMT* has 20,480 bases encoding 406 amino acids, and *BHMT2* has 20,303 bases encoding 363 amino acids. *BHMT* and *BHMT2* variants are associated with hyperhomocysteinemia and paraneoplastic polyneuropathy, respectively. Both genes are associated with spina bifida [39].

Tange et al. [99] investigated the environmental as well as maternal and infant genetic risk factors for the development of obstructive CHD. Their cohort consisted of the following CHD subtypes: TA (5%), PVS (40%), PA (4%), interrupted aortic arch (1%), HLHS (16%), Ebstein anomaly (3%), CoA (23%), and AS (8%). Intron polymorphism rs7700970 (Table 10) in *BHMT* was independently associated with an increased risk of obstructive CHD, while intron variant rs506500 (Table 10) in the same gene was associated with an increased risk of CHD only in obese mothers. Both variants are classified as benign by the ACMG, due to the high population frequencies and benign bioinformatic predictions. Considering the *BHMT2* variants, four non-coding region variants (rs1422086, rs557302, rs625879, and rs526264) (Table 10) were independently associated with an obstructive CHD, while rs557302 and rs575425 were associated with CHD in folate supplement non-user mothers [99]. All *BHMT2* variants were very common in the general population and were classified as benign by the ACMG.

### 9.2. GNMT

GNMT (EC 2.1.1.20) catalyzes the methylation of glycine by using SAM to form N-methyl glycine (sarcosine) with the concomitant production of SAH. GNMT plays a key role in the regulation of the cellular SAM levels. SAM is a major donor of methyl groups in methylation reactions, including the re-methylation of homocysteine to methionine. The *GNMT* gene is located at 6p21.1, consists of 3127 bases, and codes for 295 amino acids. Diseases associated with GNMT include GNMT deficiency and peroxisome biogenesis disorder 4B [39].

The *GNMT* gene was sparsely studied in association with CHD. Our previous study found an association of the rs10948059 variant in the maternal genome with the increased risk of CHD in the offspring [43]. However, this association was not found with the same variant in the infant genome. Nembhard et al. investigated the influence of polymorphisms in the genes of the folate methionine cycle in association with the maternal periconceptional use of SSRIs. *GNMT* variant rs11752813 (Table 11) in the infant genome was associated with the increased risk of CHD, but only in infants whose mothers were using SSRIs periconceptionally [94]. The variant (benign classification by the ACMG) is located in the promoter of the *GNMT* gene and thus influences its transcription.

### 9.3. DNMT3B

DNMT3B (EC 2.1.1.37) is a DNA methyltransferase that is thought to function in de novo methylation of the genome and is thus very important during embryonic development. The *DNMT3B* gene is located at 20q11.21. The gene has 46,975 bases and encodes 853 amino acids. Diseases associated with DNMT3B include immunodeficiency-centromeric instability-facial anomalies syndrome 1 and facioscapulohumeral muscular dystrophy 4 [39].

DNMT3B can affect the formation of CHD through two different mechanisms: direct influence on the folate/methionine pathway by the modulation of cellular SAM levels, and globally through the methylation of the fetal genome which influences the expression of numerous genes. Indeed, mRNA levels of *DNMT3B* were found to be significantly lower in CHD patients compared to healthy individuals [100].

Intron variant rs2424913 (Table 12) was more frequent in Down syndrome individuals with CHD compared to Down syndrome individuals without CHD. What is more, it was more frequent in Down syndrome children with ASD compared to those with other types of CHD [101].

Several variants in *DNMT3B* were associated with obstructive CHD, but only in cases of maternal obesity [99]. Intron variants rs6058893, rs910084, rs1883729, rs6088008, rs910085, rs4911257, rs1040555, and rs992472 (Table 12) are located in the regulatory enhancer region of the gene and probably influence its transcription rate. Using the ACMG criteria, these variants are classified as benign due to high population frequency.

### 9.4. CBS

CBS (EC 4.2.1.22) catalyzes the conversion of homocysteine to cystathionine, which is the first step in the transsulfuration pathway. The *CBS* gene is located at 21q22.3, has 23,753 bases, and encodes 551 amino acids. Mutations in CBS are associated with the inborn error of metabolism homocystinuria [39].

The rs2850144 variant (Table 13) located in the promoter of the *CBS* gene was associated with a decreased risk of CHD in general, as well as septation and conotruncal defects in the Han Chinese cohort of 2340 CHD cases and 2270 controls [102]. This is in line with the results of the functional analysis of rs2850144. In vivo measurements of *CBS* mRNA levels in human cardiac tissue and in vitro luciferase assays demonstrated the increased *CBS* gene transcription in carriers of the minor G allele [102]. Since CBS is involved in the elimination of homocysteine from the cell through transsulfuration, the carriers of the G allele might have lower levels of teratogenic homocysteine in developing cardiac tissue and thus a lower risk of CHD.

### 9.5. MAT2A

MAT2A (EC 2.5.1.6) catalyzes the production of SAM from methionine and ATP. SAM is the key methyl donor in cellular methylation processes. The *MAT2A* gene is located at 2p11.2, has 6114 bases, and codes for 395 amino acids. Mutations in this gene have been associated with the benign condition hypermethioninemia [39]. No variants in the fetal genome in this gene have been associated with CHD so far, but one study found an association of four *MAT2A* variants with lower methionine levels in mothers of CHD infants [103].

### 9.6. AHCYL1

AHCYL1 (EC 3.3.1.1) is involved in the conversion of SAH to homocysteine and adenosine. The *AHCYL1* gene is located at 1p13.3, has 38,978 bases, and encodes 530 amino acids. Diseases associated with *AHCYL1* include extrahepatic bile duct adenocarcinoma and intestinal impaction [39]. So far, no *AHCYL1* variants have been associated with CHD in humans.

## 10. Ethiopathogenic Patterns between CHD and Extracardiac Anomalies Related to Specific Genes

In this review, we found that in some instances the variants in CHD-candidate genes were associated with some extracardiac anomalies. Variants in CHD-candidate genes were most frequently related to anomalies in blood cells (including immune cells), the central neural system, intestine, skin, craniofacial region, and adrenal gland. All the above-mentioned tissues have embryonic origins in the neural crest cells. Parts of the neural system develop from several subpopulations of neural crest cells, such as cranial neural crest, vagal and sacral neural crest, trunk neural crest, and cardiac neural crest, and enteric ganglia crucial for the innervation of the colon develop from the vagal/sacral neural crest. Cartilage, bone, cranial neurons, glia, and the connective tissues of the face develop from the cranial neural crest, while adrenal glands develop from the trunk neural crest. Connective tissue and melanocytes in the skin develop from the cardiac neural crest [104]. Recently, it was found that neural crest-derived cells, which are not yet committed to the Schwann cell lineage, migrate to bone marrow alongside developing nerve fibers. These neural crest-derived cells give rise to specialized mesenchymal stem cells within the bone marrow, which contribute to the hematopoietic stem cell niche, which supports blood cell production [105].

Most extracardiac anomalies were connected to low intracellular folate levels and high homocysteine levels. As previously noted, a lack of active folate forms can have an adverse effect on embryonic development through the influence on mitosis, DNA methylation, and gene expression through folate receptors, while an excess of homocysteine can be teratogenic through increased production of reactive oxidative species, N-homocysteinylation of proteins, and antagonism of NMDA receptors. Furthermore, some CHD-candidate genes were associated with various types of cancer, probably through the folate-responsive oncogenes.

## 11. Future Research Perspectives

To date, most studies addressing the association between genetic variants in genes of folate/methionine cycles used case-control or family triad study designs, where the association of each gene with CHD was studied separately. While this approach is suitable in cases of monogenic CHD, it is less adequate in cases of polygenic forms of CHD, where the development of the anomaly is caused by multiple genes with relatively small individual contributions. This might be why the results of such studies were ambiguous since it has become clear over the years that many cases of CHD are indeed polygenic. Thus, a more suitable approach to studying genetic determinants of CHD would be to consider variants in different genes of the folate/methionine cycle in a patient, using polygenic risk score calculation. Another challenge in many of the abovementioned studies was the fact that they do not investigate the sequence of the whole gene(s) but focus only on a limited number of variants. Also, some of the genes of the folate/methionine cycle have been entirely neglected and were not studied in connection to CHD, as demonstrated earlier in this review. Future studies should therefore include targeted sequencing of all genes of the folate/methionine cycle in CHD cases and healthy controls using next-generation sequencing methods and polygenic risk score calculation. This somewhat agnostic approach will probably generate many variants and even novel variants for which the clinical significance will be unclear. In such cases, the influence of such variants on gene expression or protein product structure/function should be functionally validated in vitro on appropriate cell lines or in vivo on animal models.

The abovementioned approach of considering additive contributions of several genes to predict the probability of CHD development is harder to implement into the preconception CHD screening than in the case of the monogenic model of CHD. In addition to the higher cost of such testing, it is questionable how accurate the CHD risk calculation for an infant would be based on the polygenic risk scores of prospective parents. In contrast to monogenic CHD where risk calculations are very straightforward and based on Mendelian genetics, two high-risk parents for a polygenic trait can produce offspring with variable CHD risk levels. Thus, at the moment, preconception genetic testing would be clinically plausible only for the monogenic forms of CHD.

A total of 16 out of 31 CHD candidate genes are also pharmacogenes, although mostly with a low level of evidence. The pharmacogenes with the highest levels of evidence (i.e., 2A) in the PharmGKB database [106] are *MTHFR* and *SLC19A1*, which influence MTX toxicity and efficiency, respectively. MTX is an antifolate drug, used as an immunosuppressant and cytostatic, that uses the same transporters and metabolic enzymes as folates. Thus, the variants in genes of folate/methionine cycles can modulate its therapeutic effects. Other genes associated with MTX toxicity and efficiency (level 3) include *MTR*, *MTRR*, *DHFR*, *ABCB1*, *GGH*, *FPGS*, *FOLH1*, *MTHFD1*, *ABCC1*, *ABCC3*, and *SHMT1*. The gene associated with the highest number of drugs is the one coding for efflux transporter *ABCB1*. The most common group of drugs associated with genes of the folate/methionine cycle were anticancer drugs, including MTX, carboplatin, cisplatin, pemetrexed, 6-mercaptopurine, vincristine, doxorubicin, irinotecan, cyclophosphamide, bevacizumab, fluorouracil, and imatinib. Other drug groups were less represented and included psychoanaleptics (methylphenidate), anesthetics (sevoflurane), antiretrovirals (ritonavir), analgesics (methadone and morphine), and anti-asthmatics (montelukast). The effects of the abovementioned drugs might be influenced by folate levels or supplementation, and also genetic variants in genes of folate/methionine cycles. The identification of CHD-associated genes is important also from the therapeutic aspect, i.e., for the discovery of new drug targets for possible CHD prevention. The most obvious therapeutic approach would be to increase the re-methylation of teratogenic homocysteine to methionine by inducing an MTR-catalyzed reaction. This could be done either by supplementing the active form of folate 5-methyl-THF (methyl group donor), vitamin B12 (a cofactor of MTR), or both. In addition, the alternative re-methylation reaction catalyzed by BHMT and BHMT2 might be activated by supplementing its methyl donor betaine. At the moment, gene therapy for the monogenic forms of CHD is possible, but in the future also polygenic forms might be treatable with this approach, although this would require a complicated procedure of repairing multiple genes.

## 12. Conclusions

Lack of maternal folate supplementation in early pregnancy has been established as one of the risk factors for CHD. Genetic changes in genes coding for transporters and enzymes involved in folate/methionine cycles are potential genetic predictive markers of CHD. These could be used to identify families and individuals more vulnerable to the lack of dietary folates and/or lack of folate supplementation pre- and peri-conceptionally, resulting in higher CHD risk. However, since these variants are usually inherited in polygenic or multifactorial mode, they should be used together with other genetic markers to identify individuals and families at risk of CHD, but not as isolated diagnostic markers within families with a history of CHD.

We conclude that genetic markers in genes coding for cytosolic enzymes of folate/methionine cycles are well investigated, but more studies are needed on the influx and efflux transporters, (de)glutamation enzymes, and mitochondrial enzymes of the folate cycle.

Among genes coding for cytoplasmic enzymes of the folate cycle, *MTHFR*, *MTHFD1*, *MTR*, and *MTRR* have the strongest association with CHD, while among genes for enzymes of the methionine cycle, *BHMT* and *BHMT2* are the most prominent. Among mitochondrial folate cycle enzymes, *MTHFD2* plays the most important role in CHD formation, while *FPGS* was identified as important in the group of (de)glutamation enzymes. Among transporters, the strongest association with CHD was demonstrated for *SLC19A1*.

## Figures and Tables

**Table 1:** Genomic location of CHD candidate genes in folate/methionine cycles in humans. Candidate genes for which the association with CHD risk was documented in genetic studies on CHD patients are written in bold. ATP Binding Cassette Subfamily B Member 1 (ABCB1), solute carrier family 19 member 1 (SLC19A1), folylpolyglutamate synthase (FPGS), dihydrofolate reductase (DHFR), methylenetetrahydrofolate dehydrogenase, cyclohydrolase, and formyltetrahydrofolate synthetase 1 (MTHFD1), methenyltetrahydrofolate synthetase (MTHFS), 5-methyltetrahydrofolate-homocysteine methyltransferase (MTR), 5-methyltetrahydrofolate-homocysteine methyltransferase reductase (MTRR), methylenetetrahydrofolate reductase (MTHFR), betaine-homocysteine S-methyltransferase (BHMT), betaine-homocysteine S-methyltransferase 2 (BHMT2), glycine N-methyltransferase (GNMT), DNA methyltransferase 3 β (DNMT3B), cystathionine β-synthase (CBS), methylenetetrahydrofolate dehydrogenase 2, and methenyltetrahydrofolate cyclohydrolase (MTHFD2).

Location	Influx Folate Transporters	Efflux Folate Transporters	Enzymes Involved in Folate (De)Glutamation	Cytosolic Enzymes of the Folate Cycle	Enzymes of the Methionine Cycle	Mitochondrial Enzymes of the Folate Cycle
Chr. 1				***MTHFR*** ***MTR***	*AHCYL1*	
Chr. 2					*MAT2A*	***MTHFD2***
Chr. 3				*ALDH1L1*		
Chr. 5				***MTRR*** ***DHFR***	***BHMT*** ***BHMT2***	
Chr. 6					***GNMT***	*MTHFD1L*
Chr. 7		***ABCB1***				
Chr. 8			*GGH*			
Chr. 9			***FPGS***			
Chr. 11	*FOLR1* *FOLR2* *FOLR3*		*FOLH1*			
Chr. 12						*ALDH1L2* *SHMT2*
Chr. 14				***MTHFD1***		
Chr. 15				***MTHFS***		
Chr. 16		*ABCC1*				
Chr. 17	*SLC46A1*	*ABCC3*		*SHMT1*		
Chr. 20					***DNMT3B***	
Chr. 21	***SLC19A1***			*FTCD*	***CBS***	

**Table 4:** A variant in the *FPGS* gene that is associated with congenital heart disease in humans.

Variant ID and Type	Population Allelic Frequency (GnomAD)	ACMG Classification	Associated CHD Phenotypes	References
rs1544105 NM_004957.6: c.-2479C>T Intron	48%	Benign	LVOTO	[43]

Congenital heart disease (CHD), left ventricular outflow tract obstruction (LVOTO).

**Table 5:** Variants in the *DHFR* gene that is associated with congenital heart disease in humans.

Variant ID and Type	Population Allelic Frequency (GnomAD)	ACMG Classification	Associated CHD Phenotypes	References
rs11951910 NM_000791.4: c.243-5643A>G Intron	12%	Benign	Conotruncal	[57]
rs70991108 NM_000791.4: c.86+59_86+60insACCTGGGCGGGACGCGCCA Intron indel	51%	Benign	CHD in general	[47]

Congenital heart disease (CHD).

**Table 9:** A variant in the *MTHFS* gene that is associated with congenital heart disease in humans.

Variant ID and Type	Population Allelic Frequency (GnomAD)	ACMG Classification	Associated CHD Phenotypes	References
rs12438477 NM_006441.4: c.379+3152G>T Intron	43%	Benign	CHD in general	[94]
			Conotruncal	[95]

Congenital heart disease (CHD).

**Table 10:** Variants in the *BHMT* and *BHMT2* genes associated with congenital heart disease in humans.

Variant ID and Type	Population Allelic Frequency (GnomAD)	ACMG Classification	Associated CHD Phenotypes	References
*BHMT*				
rs7700970 NM_001713.3: c.34-266C>T Intron	32%	Benign	Obstructive CHD	[99]
rs506500 NM_001713.3: c.167-745T>C Intron	72%	Benign	Obstructive CHD	[99]
*BHMT2*				
rs1422086 NM_017614.5: c.167-327C>A Intron	53%	Benign	Obstructive CHD	[99]
rs557302 NM_017614.5: c.450+633A>G Intron	44%	Benign	Obstructive CHD	[99]
rs625879 NM_017614.5: c.1010+2010A>C Intron	53%	Benign	Obstructive CHD	[99]
rs526264 NM_017614.5: c.451-222A>T Non-coding transcript exon	53%	Benign	Obstructive CHD	[99]
rs575425 NM_017614.5: c.*5515A>G 3 prime UTR variant	62%	Benign	Obstructive CHD	[99]

Congenital heart disease (CHD), the asterisk (*) at rs575425 indicates that the mutation is located in the 3 prime UTR region.

**Table 11:** A variant in the *GNMT* gene that is associated with congenital heart disease in humans.

Variant ID and Type	Population Allelic Frequency (GnomAD)	ACMG Classification	Associated CHD Phenotypes	References
rs11752813 NM_018960.6: c.-489C>G Regulatory, promoter	40%	Benign	CHD in general	[94]

Congenital heart disease (CHD).

**Table 12:** Variants in the *DNMT3B* gene associated with congenital heart disease in humans.

Variant ID and Type	Population Allelic Frequency (GnomAD)	ACMG Classification	Associated CHD Phenotypes	References
rs2424913 NM_006892.4: c.307-49C>T Intron	56%	Benign	CHD in general	[101]
			ASD	[101]
rs6058893 NM_006892.4 Intron	52%	Benign	Obstructive CHD	[99]
rs910084 NM_006892.4: c.921+151C>T Intron	39%	Benign	Obstructive CHD	[99]
rs1883729 NM_006892.4: c.-6-4931G>A Intron	56%	Benign	Obstructive CHD	[99]
rs6088008 NM_006892.4: c.1127-355A>G Intron	39%	Benign	Obstructive CHD	[99]
rs910085 NM_006892.4: c.1252+13T>G Intron	49%	Benign	Obstructive CHD	[99]
rs4911108 NM_006892.4: c.654+54A>G Intron	41%	Benign	Obstructive CHD	[99]
rs4911257 NM_006892.4: c.-6-8550T>C Intron	43%	Benign	Obstructive CHD	[99]
rs1040555 NM_006892.4: c.922-231A>T Intron	50%	Benign	Obstructive CHD	[99]
rs992472 NM_006892.4: c.1490+164G>T Intron	49%	Benign	Obstructive CHD	[99]
rs4911107 NM_006892.4: c.433-45G>A Intron	51%	Benign	Obstructive CHD	[99]
rs2889703 NM_006892.4: c.814-223C>A Intron	49%	Benign	Obstructive CHD	[99]

Congenital heart disease (CHD), atrial septal defect (ASD).

**Table 13:** A variant in the *CBS* gene that is associated with congenital heart disease in humans.

Variant ID and Type	Population Allelic Frequency (GnomAD)	ACMG Classification	Associated CHD Phenotypes	References
rs2850144 NM_000071.3: c.-1181G>C 5 prime UTR variant, promoter	68%	Benign	CHD in general	[102]
			Septation defects	[102]
			Conotruncal	[102]

Congenital heart disease (CHD).

## Data Availability

Data sharing is not applicable.
